# Mesenchymal Stem Cell (MSC)-Based Drug Delivery into the Brain across the Blood–Brain Barrier

**DOI:** 10.3390/pharmaceutics16020289

**Published:** 2024-02-18

**Authors:** Toshihiko Tashima

**Affiliations:** Tashima Laboratories of Arts and Sciences, 1239-5 Toriyama-cho, Kohoku-ku, Yokohama 222-0035, Japan; tashima_lab@yahoo.co.jp

**Keywords:** the blood–brain barrier, mesenchymal stem cell-based drug delivery, transmembrane drug delivery

## Abstract

At present, stem cell-based therapies using induced pluripotent stem cells (iPSCs) or mesenchymal stem cells (MSCs) are being used to explore the potential for regenerative medicine in the treatment of various diseases, owing to their ability for multilineage differentiation. Interestingly, MSCs are employed not only in regenerative medicine, but also as carriers for drug delivery, homing to target sites in injured or damaged tissues including the brain by crossing the blood–brain barrier (BBB). In drug research and development, membrane impermeability is a serious problem. The development of central nervous system drugs for the treatment of neurodegenerative diseases, such as Alzheimer’s disease and Parkinson’s disease, remains difficult due to impermeability in capillary endothelial cells at the BBB, in addition to their complicated pathogenesis and pathology. Thus, intravenously or intraarterially administered MSC-mediated drug delivery in a non-invasive way is a solution to this transendothelial problem at the BBB. Substances delivered by MSCs are divided into artificially included materials in advance, such as low molecular weight compounds including doxorubicin, and expected protein expression products of genetic modification, such as interleukins. After internalizing into the brain through the fenestration between the capillary endothelial cells, MSCs release their cargos to the injured brain cells. In this review, I introduce the potential and advantages of drug delivery into the brain across the BBB using MSCs as a carrier that moves into the brain as if they acted of their own will.

## 1. Introduction

**Figure 1:**
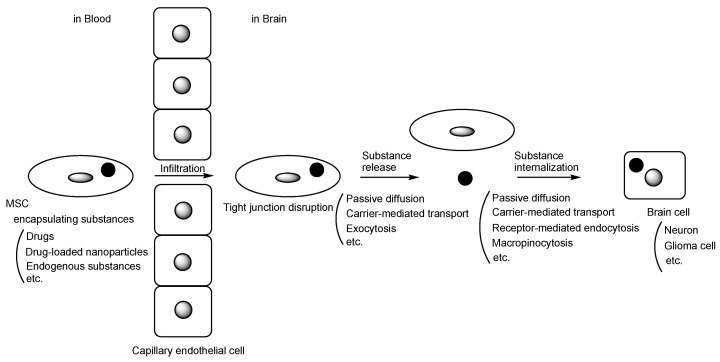
The pathway of mesenchymal stem cell (MSC)-based drug delivery into the brain across the blood–brain barrier. A black circle represents substances such as drugs, drug-loaded nanoparticles, or endogenous compounds. A gray circle or a gray ellipse represents a cell nucleus.

Stem cells offer a wide variety of possibilities for new medical treatments due to their ability to develop into many different cell types. Mesenchymal stem cells (MSCs), also known as mesenchymal stromal cells, have potential applications in therapeutic contexts due to their multilineage differentiation capacity and anti-inflammatory properties. Intriguingly, MSCs can serve as drug carriers, homing to specific target sites. In drug research and development, incorrect distribution and membrane impermeability pose serious challenges. Therefore, molecular target drugs, such as monoclonal antibody drugs, are being developed to address selectivity issues and are currently available for clinical use. Even molecular target drugs face challenges, including impermeability through passive diffusion due to size and hydrophilicity. Monoclonal antibodies, however, are utilized as a vector for monoclonal antibody-drug conjugates to traverse the blood–brain barrier (BBB) via receptor-mediated transcytosis in capillary endothelial cells through the transcellular route [1,2]. The BBB is primarily composed of (i) tight junctions between capillary endothelial cells facilitated by adhesion molecules like claudin, (ii) the hydrophobic lipid bilayer membrane of capillary endothelial cells, (iii) biological efflux transporters such as multiple drug resistance 1 (MDR1) (P-glycoprotein (P-gp)) on the apical membrane of capillary endothelial cells, and (iv) the support provided by pericytes and astrocytes. Furthermore, the development of drugs for the treatment of Parkinson’s disease (PD) and Alzheimer’s disease (AD) [3,4] in the central nervous system continues to face unmet medical needs, primarily due to impermeability caused by the BBB, in addition to their complicated pathogenesis and pathology. Therefore, utilizing MSCs for delivery could serve as a solution for highly selective and efficient delivery to disease sites, especially the injured or damaged brain. Indeed, drug delivery utilizing MSCs is currently under development. It is true that sone reviews focusing on the drug delivery using MSCs-derived exosomes are reported [5,6,7]. However, MSC-based drug delivery into the brain across the BBB can be considered a more dynamic strategy, particularly compared to isolated exosomes. Therefore, in this perspective review, I introduce the possibilities and approaches for drug delivery into the brain across the BBB using MSCs (Figure 1).

**Figure 2:**
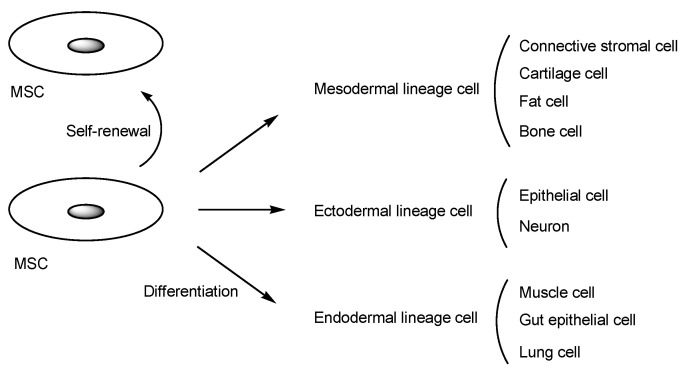
Schematic diagram illustrating mesenchymal stem cell (MSC) differentiation. A gray ellipse represents a cell nucleus.

MSCs are pluripotent cells with self-renewal and differentiation potential. They can differentiate into mesodermal lineage cells, such as connective stromal cells, cartilage cells, fat cells, and bone cells; ectodermal lineage cells, such as epithelial cells and neurons; or endodermal lineage cells, such as muscle cells, gut epithelial cells, and lung cells (Figure 2) [8]. MSCs can be isolated from a variety of tissues. Intriguingly, MSCs sense signals from injured tissues or areas of disease and migrate to these locations to promote recovery. Moreover, MSCs play a vital role in regulating the cellular microenvironment, leading to immunomodulation, hematopoietic support, tissue repair, and tissue regeneration through interactions with various cells, including macrophages, neutrophils, cancer cells, hematopoietic stem cells, and others, mediated by cytokines [9]. Contrarily, MSC differentiation fates are influenced by the cellular microenvironment. It is known that MSCs secrete not only cytokines but also exosomes [10]. Therefore, the potential of MSCs is immeasurable.

## 2. Discussion

### 2.1. Human MSCs for Regenerative Medicine

Numerous clinical trials involving human MSCs for regenerative medicine are currently underway (Table 1). Some of these trials have already advanced to the pharmaceutical market. Representative cases are briefly introduced here [11], because the main theme of this perspective review is drug delivery. Other information can be obtained from references [12,13]. (i) Sutéramikku, autologous bone marrow-derived human MSCs (English name unknown, identified by the investigational drug code STR01 [14]), received conditional approval in 2018 in Japan for the improvement of neurological symptoms and functional impairment associated with spinal cord injury, marking a global milestone. Nevertheless, the approval, which was contingent on a study involving 13 patients in the active drug group, has generated controversy [15,16]. It is worth noting that the medications were administered promptly after undergoing the highest level of scrutiny mandated by modern scientific standards, laws, and regulations [17]. This medication is currently available clinically. Regulatory frameworks for new modalities should be established, aiming not only to offer patients the latest medical care, but also to prevent medical accidents. (ii) Temcell HS [18], allogeneic bone marrow-derived human MSCs, received approval for the treatment of acute graft-versus-host disease in Japan in 2015. (iii) Alofisel (darvadstrocel) [19], allogeneic bone marrow-derived human MSCs, received approval for the treatment of complex perianal fistula in adults with Crohn’s disease in the European Union in 2017. (iv) SB623 (vandefitemcel) [20], allogeneic bone marrow-derived human MSCs, successfully completed a phase 2 clinical trial (STEMTRA trial) with positive results for traumatic brain injury. As a result, it was submitted for early approval with conditions in Japan in March 2022. (v) FF-31501, autologous bone marrow-derived human MSC, is currently undergoing a phase 3 clinical trial for meniscal injury in Japan, which commenced in February 2023. (vi) CYP-004, iPSC-derived human MSCs, have been undergoing a phase 3 clinical trial for osteoarthritis (ACTRN12620000870954) in Australia since November 2020. (vii) MutiStem (HLCM051), allogeneic bone marrow-derived human MSCs, underwent a phase 3 clinical trial for ischemic cerebral infarction (MASTERS-2 trial, NCT03545607) in the USA from July 2018 to June 2023. (viii) Rexlemestrocel-L, allogeneic bone marrow-derived human MSCs, underwent a phase 3 clinical trial for lower back pain (MSB-DR003 trial, NCT02412735) in the USA from March 2015 to June 2021. (ix) Remestemcel-L, an allogeneic bone marrow-derived human MSC product, underwent a phase 3 clinical trial for acute respiratory distress syndrome. This is an manufactured MSC product equivalent to Temcell HS. (x) gMSC1, allogeneic synovial membrane-derived human MSC, is currently undergoing a phase 3 clinical trial for knee cartilage damage since November 2017 in Japan.

The preparation of human MSCs and the clinical use of MSC-secretome are strictly subject to current good manufacturing practice (cGMP) standards [21]. MSCs from other donors might pose immunological challenges for allogeneic use, because they are not obtained from patient’s own cells for autologous use. Thus, hypoimmunogenicity, low immunogenicity, immune tolerance should be necessary when allogeneic MSCs are used. Nonetheless, human MSCs are immunoprivileged because they possess very low levels of major histocompatibility complex (MHC) class I and no MHC class II, which are insufficient to induce activation of allogeneic lymphocytes [22].

Nevertheless, it is probable that human MSCs as carriers for drug delivery have not yet received clinical approval.

### 2.2. The Potential of MSC-Mediated Drug Delivery

It is known that the majority of intravenously administered rat MSCs are trapped in the lungs through the pulmonary first-pass effect. This is attributed to the cell size and the expression of adhesion molecules targeting receptors on the surface of pulmonary capillary endothelial cells, as observed in in vivo tests using rats [23]. This phenomenon might pose a relatively serious problem for MSC delivery, particularly into the brain. Moreover, when rat MSCs (with an average diameter of 23 μm) were bolused into the ipsilateral common iliac artery in rats, they spread out on the luminal side of the vessel and subsequently localized in a perivascular niche within 72 h [24]. In other words, the half-life of rat MSCs was roughly assumed to be around 36 h by approximating the attenuation curve as a straight line in this case. Generally, the diameter of human capillaries is approximately 5–20 μm [25]. Human red blood cells, with a disk diameter of 6.2–8.2 μm and a thickness of 2–2.5 μm at the thickest part [26], can pass through capillaries. There are various opinions regarding the size of MSCs. Nonetheless, the volume of rat MSCs could be roughly estimated at approximately 3000 μm^3^, although there is variety in size [23]. Thus, their diameter is calculated to be approximately 18 μm when they are considered spherical. On the other hand, the average diameter of bone marrow-derived human MSCs cultured in low serum/serum-free media (SFM)/Xeno-free media (XFM) ranged from 16.02 to 19.20 μm [27]. MSCs can change their shape to a spindle-like shape. Therefore, while most MSCs might be small enough to pass through capillaries, it is thought that they move slowly in narrow regions and bind to the surface of capillary endothelial cells due to adhesion molecules interacting with cell surface receptors, and possibly due to wall fluid shear stress (FSS) (approximately 0.4 Pa) pushing them against the cell surface [28]. Anatomically, the surface area of human adult pulmonary capillaries is approximately 50–70 m^2^ [29], whereas that of human adult brain capillaries is approximately 12–18 m^2^ [30]. The pulmonary capillary mesh cannot be bypassed given its scale in the normal course. Systematically, all intravenously administered substances pass through pulmonary capillaries before reaching brain capillaries. It is preferable to administer MSCs through carotid artery injection or intranasal administration to avoid the pulmonary first-pass effect, based on the biological and physical systematic structures regulated by structuralism advocated by Dr. Lévi-Strauss [31,32]. Nose-to-brain routes through intranasal administration will be described at another time. Human MSCs, acting as both carriers and vectors, can store various pharmaceutical agents as cargo, such as drugs or nanoparticles containing drugs, within the cells or load them on the surface of the cells [33].

The suspension of human MSCs and various numbers of microcapsules was fixed in 2.5% glutaraldehyde and then centrifuged at 1500 g. The resulting precipitates were MSCs containing microcapsules. The cellular motility of MSCs was reduced as the number of loaded microcapsules increased. MSCs with microcapsules at a ratio of 1:10 demonstrated greater migration than those at 1:20 and 1:45, respectively, but still showed lower migration than the control group with no microcapsules. Morphologically, MSCs changed their shape from a spindle-like shape to an irregular shape, especially with microcapsules at a greater ratio of 1:20 [34]. Thus, it is suggested that MSCs should not be loaded with too many microcapsules to maintain migration ability and cell morphology.

### 2.3. The Implementation of MSC Delivery into the Brain across the BBB

#### 2.3.1. Glioma

Brain cancers are characterized by cancerous growth within the brain and are broadly categorized into various types, including glioma, metastatic brain tumors, medulloblastoma, malignant lymphoma, germ cell tumors, meningioma, hypophyseal adenoma, and neurilemmoma. Glioma [35] is the most common type of CNS neoplasm and originates from glial cells rather than metastasizing. Specifically, glioblastomas are highly malignant gliomas. Anti-brain cancer drugs face challenges in entering the brain due to the BBB, leading to unmet medical needs due to the lack of effective pharmaceutical agents. Therefore, an innovative drug delivery system should be established promptly.

MSCs can migrate to cancer foci due to cytokines secreted from the tumor microenvironment along a chemoattractant gradient [33]. On the other hand, metastasis from peripheral cancer tissues into the brain is gradually accomplished based on the ‘seed and soil’ theory proposed by Dr. Stephen Paget [36]. Exosomes released into the bloodstream from peripheral cancer cells fused with the membrane of capillary endothelial cells at the BBB, eventually disrupting the tight junctions. As a result, cancer cells migrated to the brain through the BBB disruption [37]. Therefore, MSCs could migrate to cancer foci in the brain across the BBB, similar to the BBB transmigration by cancer cells from peripheral cancer tissues in the metastasis process.

The BBB is significantly altered by brain cancer cells. During this process, brain cancer cells secrete vascular endothelial growth factors (VEGFs) that induce angiogenesis, cause the disappearance of astrocyte endfeet, and disrupt tight junctions to form fenestration. However, transport of substances between the bloodstream and the brain cancer stroma is regulated. This situation is referred to as the blood–tumor barrier. Anticancer drugs still face difficulty in entering the brain due to the blood–tumor barrier, even though there are fenestrations between the capillary endothelial cells. Hydrophilic nutrients and essential materials are delivered to the brain cancer stroma and cancer cells through the paracellular fenestration pathway and transcellular pinocytosis [38,39]. Thus, cytokines secreted from the tumor microenvironment in the brain might leak into the bloodstream through the fenestrations of the capillary endothelial cells at the BBB. It is believed that such cytokines might attract migrating cancer cells or MSCs into the brain.

**Figure 3:**
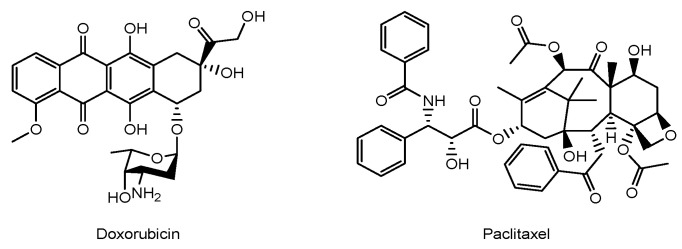
The structures of anti-cancer drugs.

It is true that low-molecular-weight anti-cancer agents are effective against glioma, but they cannot cross the BBB due to excretion by MDR1. Thus, MSC-mediated drug delivery into the brain across the BBB is one of the solutions. MSCs loaded with anti-cancer drugs might be a promising tool for delivery into brain cancer cells. MSCs might be relatively tolerant to commonly used anti-cancer agents such as doxorubicin or paclitaxel (PTX) (Figure 3), probably due to differences in cell division speed. In the case of MSC damage, the use of nanoparticles encapsulating anti-cancer drugs might transiently avoid such cell damage, particularly within 72 h until their localization in a perivascular niche. Some of the drugs might be released from the intracellular nanoparticles to the cytosol via passive diffusion or from the extracellular nanoparticles.

(i) MSCs containing silica nanorattles encapsulating doxorubicin tracked down U251 glioma tumor cells more efficiently and enhanced tumor cell apoptosis in in vivo assays using rodents compared to doxorubicin alone or silica nanorattles encapsulating doxorubicin alone without using MSCs [40]. (ii) PTX-encapsulated hyaluronic acid-poly (D,L-lactide-co-glycolide) polymeric micelles (PTX/HA-PLGA micelles) (141.2 ± 0.5 nm in diameter) were efficiently internalized through receptor-mediated endocytosis, utilizing CD44 as a receptor in CD44 overexpressing MSCs. Both clathrin- and caveolae-mediated endocytosis pathways were involved in this internalization. MSCs exhibited tolerance to PTX due to the localization of PTX/HA-PLGA micelles in endosomes. MSCs containing PTX/HA-PLGA micelles demonstrated anti-glioma efficacy following contralateral injection in an in vivo assay using C6 glioma-bearing rats. PTX-encapsulated micelles were exocytosed into the brain and subsequently entered glioma cells via receptor-mediated endocytosis utilizing CD44. Finally, PTX was released from the micelles in endosomes and penetrated the cytosol through the endosomal membrane via passive diffusion [41].

Oncolytic viruses selectively infect cancer cells and eventually lyse them without infecting healthy cells [42]. In fact, glioma cells were destroyed by oncolytic viruses [43]. Teserpaturev (G47Δ, Delytact), a third generation (triple-mutated) recombinant oncolytic herpes simplex virus type 1, was conditionally approved for malignant glioma in June 2021 in Japan [44]. However, oncolytic viral agents were typically administered into the brain through stereotactic brain surgery. Oncolytic virotherapy for glioma tumors should be conducted in a non-invasive manner to reduce the burden on patients. Therefore, delivering oncolytic viruses into the brain using MSCs as a carrier and vector is a useful approach for glioma treatment. (iii) Human umbilical cord blood-derived MSCs loaded with a novel oncolytic adenovirus carrying interleukin (IL)-24 and/or endostatin demonstrated significantly greater antitumor effects in a xenograft model of glioma [45]. IL-24, a cytokine belonging to the IL-10 family, inhibited the growth of tumor cells and induced tumor-specific apoptosis [46]. Endostatin, a fragment of collagen XVIII, inhibits angiogenesis [47]. A synergistic anti-cancer effect was exhibited. (iv) Moreover, the oncolytic virus, CRAd.S.pK7, encapsulated within MSCs, entered and replicated in diffuse intrinsic pontine glioma using preclinical xenografted mouse models. Oncolytic virus-loaded MSCs, when combined with radiotherapy, exhibited prolonged survival compared to either therapy alone in mice bearing brainstem DIPG xenografts [48].

The modification of MSCs, such as overexpressing CXC chemokine receptor 4 (CXCR4) using a retroviral vector, is an improvement for homing to the tumor [49]. CXCR4 is a G-protein-coupled seven-transmembrane receptor on the cancer cell and is highly expressed in MSCs within the bone marrow, enabling MSCs to migrate to CXCR4 ligands at injured sites. Stromal cell-derived factor-1α (SDF-1α) (CXCL12) is a chemokine serving as a CXCR4 ligand that facilitates MSC tropism to tumors such as glioma. Additionally, the CXCR4/SDF-1α axis is highly relevant in cell recruitment during CNS injury [50]. Thus, strategies for actively homing MSCs to cancers might be feasible.

#### 2.3.2. Parkinson’s Disease (PD)

PD [51] is a representative neurodegenerative disease caused by the loss of dopaminergic neurons in the nigrostriatal pathway due to the extracellular aggregation of α-synuclein, forming Lewy bodies. It manifests with symptoms such as motor dysfunction, including bradykinesia, tremor, and rigidity. Therefore, there is an expectation for innovative therapeutic agents. In general, nucleic acid-based drugs, such as antisense oligonucleotides, small interfering RNA (siRNA), and microRNA (miRNA), are enzymatically unstable in the serum due to nucleases. Endogenous miRNAs released from cells are protected within exosomes in the bloodstream. Similarly, MSCs can be used as carriers to protect delicate cargos such as RNAs. Nonetheless, MSC-derived exosomes or extracellular vesicles are likely to be sufficient for delivering miRNAs, such as miR-181a-2-3p, into the brain [52]. In PD treatment, MSCs are often used as regenerative medicine to differentiate into dopaminergic neurons [53]. However, genetically engineered MSCs are developed as an alternative approach for use as carriers. (i) Interestingly, MSCs encoding three critical genes for dopamine synthesis restored striatal dopamine levels and ameliorated motor function in PD rats [54]. (ii) MSCs primed with α-synuclein elicited neuroprotective effects by enhancing autophagy-mediated α-synuclein clearance. The expression of autophagy-regulating miRNAs, such as miR-376-3p, was increased in MSCs preliminarily primed with α-synuclein and subsequently packaged into exosomes derived from primed MSCs. Ultimately, MSCs primed with α-synuclein demonstrated more pronounced neuroprotective effects on dopaminergic neurons by inducing autophagy and lysosome activity in an in vivo assay using α-synuclein-overexpressing mice compared to naïve MSCs [55]. (iii) Matrix metalloproteinase-2 (MMP-2) derived from MSCs cleaved α-synuclein fibrils into smaller insoluble and oligomeric forms in the brain of a mouse PD model. The human MSCs used were not genetically manipulated. Conversely, MMP-2 knockdown MSCs through siRNA increased the intensity of α-synuclein aggregates in SH-SY5Y cells preincubated with α-synuclein, compared to normal MSCs [56]. Moreover, novel antisense oligonucleotides targeting mRNA coding for α-synuclein have been developed. Amido-bridged nucleic acid (AmNA)-modified antisense oligonucleotides targeting mRNA coding for α-synuclein demonstrated distribution to various brain areas in an in vivo assay through intrathecal administration using PD model mice, in the absence of any carrier or conjugation [57]. Is it possible to transport these antisense oligonucleotides more efficiently by using MSCs as a carrier to target the PD brain?

#### 2.3.3. Alzheimer’s Disease (AD)

AD [58] is also a representative neurodegenerative disease caused by the loss of neurons due to the aggregation of amyloid β (Aβ), forming extracellular oligomers, protofibrils, and amyloid fibrils, and tau aggregation, forming intracellular neurofibrillary tangles (NFTs). It presents with symptoms such as memory loss, mild cognitive impairment, and dementia. Thus, innovative therapeutic agents are expected, although anti-Aβ antibodies have recently been launched in the pharmaceutical market. The anti-Aβ monoclonal antibody aducanumab [59] was approved by the Food and Drug Administration (FDA) in 2021. The anti-Aβ protofibril monoclonal antibody lecanemab [60] was approved by the FDA in 2023. Furthermore, the anti-Aβ monoclonal antibody donanemab [61] completed a phase 3 clinical trial with positive results for early AD in 2023 (NCT04437511). This strategy targeting Aβ is based on the amyloid hypothesis. However, there are several hypotheses regarding the onset and progression of AD due to the complex pathogenesis mechanism. It is implied that tau-derived NFTs are more closely correlated with AD pathogenesis than Aβ-derived senile plaques [3]. Although Aβ pathology and tau pathology initially proceeded independently, it is likely that Aβ pathology enhances the spreading of tau pathology at a certain point in the progression of AD symptoms [3]. In AD treatment, MSCs are often used as a regenerative medicine to differentiate into neurons or Schwann cell-like cells [62]. It has been suggested that neuroinflammation triggered by activated microglial cells plays a key role in the pathogenesis of AD [63]. Moreover, it is well-known that IL-10 deficiency exacerbates inflammation-induced tau pathology [64]. Thus, the use of IL-10 is considered effective for AD.

(i) Wharton’s Jelly-derived MSCs improved spatial learning and alleviated memory decline after intravenous transplantation in the neuropathology and memory deficits in amyloid precursor protein (APP) and presenilin-1 (PS1) double-transgenic mice. Wharton’s Jelly-derived MSCs increased the expression of IL-10, resulting in reduced microglial activation. The expressions of pro-inflammatory cytokines such as IL-1β and TNFα were decreased [65]. (ii) Furthermore, transplantation of MSCs via the tail vein improved spatial memory in the Morris water maze test using AD model mice (APdE9). Analysis based on electron paramagnetic resonance imaging revealed that oxidative stress was suppressed, evaluating the in vivo redox state of the brain. Intriguingly, the upregulation of CD14 expression in microglia by MSCs prompted the microglial uptake of Aβ via receptor-mediated endocytosis using the TLR4/CD14 complex and its clearance in the endo-lysosomal degradation pathway in vivo. MSCs altered the microglial phenotype from M1 to M2 by Th2 cytokines such as TGFβ and IL4 secreted from MSCs, eventually suppressing the production of proinflammatory cytokines in an in vitro assay using co-cultured mouse microglial cell line MG6 with MSCs [66]. (iii) Intracerebroventricularly injected bone marrow-derived MSCs improved cognitive impairment in APP/PS1 mice as an AD model by transferring exosomal miR-146a into astrocytes. The contents in exosomes secreted from MSCs into the cerebrospinal fluid (CSF) were internalized by astrocytes through fusion with the plasma membrane. The down-regulated expression of TRAF6 and NF-κB in astrocytes suppressed astrocyte inflammation and subsequently promoted synaptogenesis [67].

Therefore, MSCs demonstrated multifunctional remedial activity in AD pathology through the supply of IL-10, Th2 cytokines, miR-146a, and other substances. The use of MSCs as carriers for nanoparticles containing biologically active substances is not widely reported, although endogenous exosomes derived from MSCs can be considered natural nanoparticles.

#### 2.3.4. Stroke

Stroke [68] is strictly divided into cerebral infarction, cerebral hemorrhage, and subarachnoid hemorrhage. Cerebral infarction [69] occurs most frequently among them, causing necrosis of brain cells due to a lack of oxygen and nutrients resulting from blood vessel occlusion in the brain. Cerebral hemorrhage [70] involves the rupture of blood vessels in the brain, often forming an intracranial hematoma that may damage brain tissue. Subarachnoid hemorrhage [71] occurs in the subarachnoid space due to the rupture of blood vessels on the surface of the brain, often causing damage to brain tissue due to highly elevated intracranial pressure. Therefore, prognosis and recurrence prevention are crucial for affected individuals to lead a comfortable life. Stem cell therapy following a stroke is receiving considerable attention for its potential in improving neuroplasticity.

(i) Reactive oxygen species (ROS) play a role in brain injury following ischemic stroke [72]. To treat ischemic stroke, oxidative stress induced by ROS should be minimized. Mitochondrial Rho-GTPase 1 (Miro1) is a calcium-sensitive adaptor protein that facilitates the axonal transport of mitochondria in neurons. Intravenously injected Miro1-overexpressed multipotent MSCs significantly improved the recovery of neurological functions in rats modeled with middle cerebral artery occlusion-induced focal ischemia. The transfer of healthy mitochondria from Miro1-overexpressed multipotent MSCs to astrocytes exposed to ischemic damage, associated with elevated ROS levels, was performed. Consequently, the recipient astrocytes restored their bioenergetics. In fact, after 2 days of co-cultivation of (a) MSCs transfected with lentiviral constructs encoding red fluorescent protein fused with a mitochondrial localization signal and (b) astrocytes transfected with a similar construct encoding green fluorescent protein with a mitochondrial localization signal, red-fluorescing mitochondria derived from MSCs were observed alongside green-fluorescing mitochondria. Green-fluorescing mitochondria were not observed within MSCs. It is suggested that the mechanism of mitochondrial transport involved the passage through tunneling nanotubes [73]. (ii) Programmed cell death-ligand 1 (PD-L1) and AKT-modified umbilical cord-derived MSCs (UMSC-PD-L1-AKT), injected into a murine stroke model to overcome the hypoxic environment of the ischemic brain through intravenous and intracarotid routes, exhibited enhanced protection of neuroglial cells from ischemic injury based on the attenuation of systemic inflammation, compared to unmodified UMSCs. CD8^+^CD122^+^IL-10^+^ regulatory T (Treg) cells were enhanced, while CD11b^+^CD80^+^ microglial/macrophages and CD3^+^CD8^+^TNF-α^+^ and CD3^+^CD8^+^IFN-α^+^ cytotoxic T cells were reduced. It is well-known that PD-L1 contributes to immune regulation. Akt activation is associated with pro-survival and anti-apoptotic effects. Akt is a serine/threonine protein kinase known as protein kinase B [74]. (iii) As an example of modified MSCs in a broad sense, three-dimensional (3D) spheroid-cultured MSCs, when intravenously injected, exhibited enhanced homing ability to the brain. Consequently, they decreased infarct volume and improved neurological function in rats that underwent middle cerebral artery occlusion and reperfusion, compared to 2D-cultured MSCs. The enhancement of the homing potential of MSCs is attributed to a reduced cell size that avoids lung entrapment and an increased expression of the chemokine receptor CXCR4. It is known that SDF-1α (CXCL12), a CXCR4 ligand, is produced by damaged neurons after cerebral ischemia. Furthermore, the anti-inflammatory properties of MSCs suppress a stimulated inflammatory response after ischemic stroke [75].

#### 2.3.5. Traumatic Brain Injury

Traumatic brain injury [76], often induced by an external force such as a violent blow, can result in cognitive dysfunction due to severe brain damage. (i) Indeed, in traumatic brain injury rats, genetically engineered MSCs overexpressing IL-10 significantly reduced the number of dead cells in the cortex and hippocampus three weeks after transplantation, compared to MSCs alone. It was suggested that IL-10 exhibited neuroprotective effects through its anti-inflammatory properties, suppressing the expression of various pro-inflammatory cytokines [77]. (ii) Brain-derived neurotrophic factor (BDNF) is involved in regulating the neurogenesis process through its binding to tropomyosin receptor kinase B (TrkB), which serves as a BDNF receptor on brain cells. Engineered MSCs overexpressing BDNF were intracerebroventricularly administered directly into the left lateral ventricle of the brain, ultimately improving the neurological and cognitive functions of traumatic brain injury rats in the Morris water maze test compared to a negative control. Thus, it was suggested that engineered MSCs secreted significantly more BDNF than naïve MSCs [78]. (iii) Fibroblast growth factor 21 (FGF21) is a type of secreted growth factor that acts as a metabolic regulator by binding to fibroblast growth factor receptors. MSCs overexpressing FGF21, administered through a hole drilled on the contralateral side of the injured hemisphere, enhanced neurogenesis in a mouse model of traumatic brain injury compared to a negative control. Thus, it was suggested that FGF21 released from MSCs induced neurogenesis [79].

#### 2.3.6. Amyotrophic Lateral Sclerosis (ALS)

ALS [80] is a progressive neurodegenerative disease that causes muscle weakness due to the disorder of the motor neuron system. The causes of ALS are still unknown, although more than 30 causative gene mutations including Cu/Zn-superoxide dismutase (SOD1) have been discovered. Many clinical trials using MSCs are performed for the recovery of ALS [12]. These are not likely to be conducted as a carrier to aim the delivery or expression of specific compounds in the brain, although a type of effects including neuroinflammation or mitochondrial transfer are expected in addition to regenerative action [81]. Nonetheless, such accidental cargo might be improved. Tofersen, an oligonucleotide medicine targeting mRNA derived from SOD1 mutation, was clinically approved by the FDA in April, 2023 for the treatment of ALS associated with a mutation in the SOD1 gene [82]. Thus, MSC-mediated tofersen delivery into the brain can be a promising strategy due to enzymatic instability of oligonucleotide medicines and the impermeability across the BBB.

#### 2.3.7. Multiple Sclerosis

**Figure 4:**
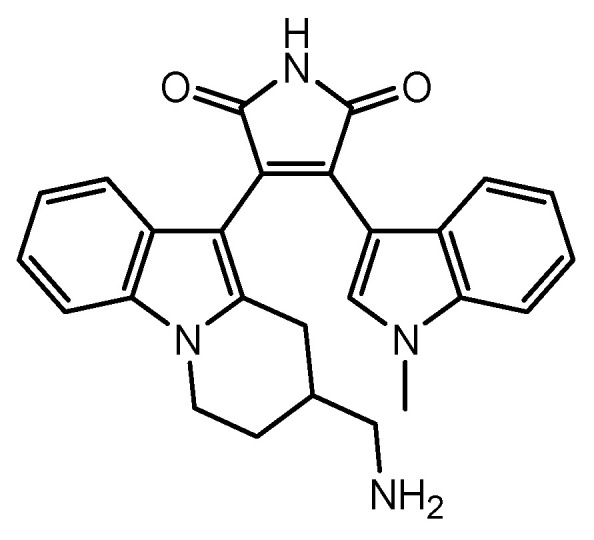
The structure of Ro-31-8425.

Multiple sclerosis [83] is an immune-mediated inflammatory disease that affects the brain and spinal cord due to attacks mistaken by the body’s immune system. The causes of multiple sclerosis are also still unknown, which are suggested to be a combination of genetic, immunologic, and environmental factors. Many clinical trials using MSCs are performed for the recovery of multiple sclerosis [12]. It turned out that MSCs were utilized to prevent circulating immune cells from crossing the BBB as disease-modifying therapies [84]. However, these processes are not considered as the drug delivers into the brain across the BBB using MSCs. Immunomodulatory strategies are thought to be effective on multiple sclerosis. (i) Ro-31-8425 is a cell-permeable, low-molecular weight ATP-competitive kinase inhibitor that inhibits human neutrophil superoxide generation (Figure 4) [85]. Systemically administered Ro-31-8425-loaded MSCs were more effective than MSCs alone or free Ro-31-8425 alone in the experimental autoimmune encephalomyelitis (EAE) mouse model of multiple sclerosis. Nonetheless, the serum level of Ro-31-8425 of Ro-31-8425-loaded MSCs was higher than Ro-31-8425 alone [86]. Accordingly, it was uncertain whether Ro-31-8425-loaded MSCs delivered their cargo into the brain across the BBB or not. Moreover, ocrelizumab, an anti-CD20 monoclonal antibody, was clinically approved by the FDA for the treatment of primary progressive multiple sclerosis (PPMS) disease progression in March 2017 [87]. Generally, monoclonal antibodies cannot cross the BBB via passive diffusion. Thus, MSC-mediated ocrelizumab delivery into the brain might enhance the activity.

### 2.4. MSCs as a Carrier of Nanoparticles

**Figure 5:**
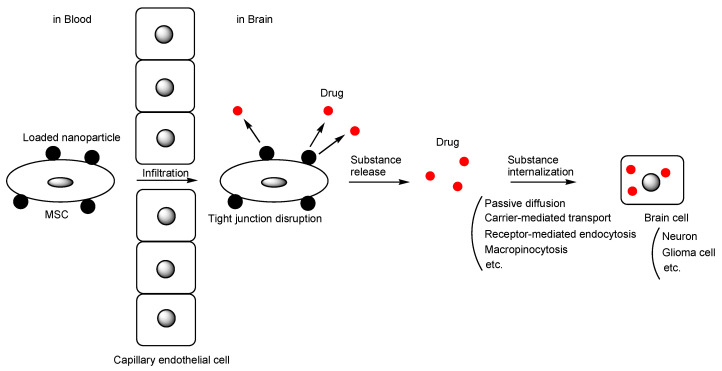
The pathway of nanoparticle loaded-mesenchymal stem cell (MSC)-based drug delivery into the brain across the blood–brain barrier. A black circle means nanoparticles containing drugs shown in a red circle. Released drugs from nanoparticles in the brain are internalized into brain cells.

It appears that the technology to use MSCs as carriers for nanoparticles containing biologically active substances has not been extensively developed, except in the field of brain tumors, such as doxorubicin- or PTX-encapsulated nanoparticles. The method of introducing nanoparticles onto the surface of MSCs (Figure 5) might be technologically easier than their internalization into MSCs, as demonstrated by neutrophils externally bound to functional microparticles entering the brain across the BBB for the treatment of glioblastoma [88]. Allogeneic sources are suitable for the preparation of modified MSCs to conduct sudden clinical use without any time difference. Furthermore, the surface modification on MSCs using nanoparticles is ideal for such sudden use due to easiness and high quality. Alternatively, internalizing cargo substances into exosomes produced by MSCs under the regulation of exosome biogenesis might be a solution, instead of using exosomes derived from isolated MSCs [5,6,7]. Otherwise, isolated MSCs-derived exosomes containing cargo substances might be reintroduced into the MSCs. The potential for MSC therapy will expand. Moreover, human artificial chromosomes are non-integrating chromosomal gene delivery vectors used for gene and cell therapies Duchene muscular dystrophy and for cancer therapy. Established MSCs with human artificial chromosomes might play an important role in MSC-based drug delivery [89], although human artificial chromosomes are a technology different from nanoparticles.

## 3. Conclusions

Stem cells are gaining attention in the field of regenerative medicine, with MSCs being particularly noteworthy due to their easy obtainability from various tissues. Moreover, MSCs can serve as drug carriers that can home accurately and autonomously to target sites, including the brain, by traversing the BBB through transiently formed fenestrations between capillary endothelial cells. Cargos delivered by MSCs are divided into materials artificially included in advance, such as doxorubicin, and expected protein expression products of genetic modification, such as interleukins, including IL-10. Success has been observed in both cases, as mentioned above. Various types of materials are delivered to the target sites without serious off-target side effects (Table 2). Placing nanoparticles on the surface of MSCs is more realistic [88] than encapsulating them within MSCs, due to technical problems and material trajectory based on structuralism. Additionally, MSCs should be administered via carotid artery injection to avoid the pulmonary first-pass effect.

MSCs move to the injured sites as if acting of their own will, whereas compounds such as low-molecular-weight drugs, antibodies, nutrients, poisons, and waste materials move spontaneously and probabilistically to the target sites, completely based on the biological and physical machinery system regulated by structuralism. Nevertheless, it is true that MSC movement is subject to the biological and physical machinery system to some extent, but MSC therapy could have more immense possibilities, not only in the role of regenerative medicine, but also as a potent drug carrier than conventional medicine. Therefore, MSC therapy will rapidly evolve to deliver innovative medical care to patients in various roles.

## Figures and Tables

**Table 1:** Numerous clinical trials utilizing human mesenchymal stem cells (MSCs) in regenerative medicine.

#	Name	MSC Type	Diseases	Status	References
1	Sutéramikku	Autologous bone marrow-derived human MSCs	Spinal cord injury	Launched	[14,15,16]
2	Temcell HS	Allogeneic bone marrow-derived human MSCs	Acute graft-versus-host disease	Launched	[18]
3	Alofisel (darvadstrocel)	Allogeneic bone marrow-derived human MSCs	Complex perianal fistula in adults with Crohn’s disease	Launched	[19]
4	SB623 (vandefitemcel)	Allogeneic bone marrow-derived human MSCs	Traumatic brain injury	Launched	[20]
5	FF-31501	Autologous bone marrow-derived human MSCs	Meniscal injury	Phase 3 clinical trial	-
6	CYP-004	iPSC-derived human MSCs	Osteoarthritis	Phase 3 clinical trial (ACTRN12620000870954)	-
7	MutiStem (HLCM051)	Allogeneic bone marrow-derived human MSCs	Ischemic cerebral infarction	Phase 3 clinical trial (MASTERS-2 trial, NCT03545607), finished in2023	-
8	Rexlemestrocel-L	Allogeneic bone marrow-derived human MSCs	Lower back pain	Phase 3 clinical trial (MSB-DR003 trial, NCT02412735), finished in 2021	-
9	Remestemcel-L	Allogeneic bone marrow-derived human MSCs	Acute respiratory distress syndrome	Phase 3 clinical trial, finished	-
10	gMSC1	Allogeneic synovial membrane-derived human MSCs	Knee cartilage damage	Phase 3 clinical trial	-

**Table 2:** The introduced applications of drug delivery into the brain using MSCs as a carrier.

#	Formulation	Diseases	Cargo	Status	References
1	MSCs containing silica nanorattle encapsulating doxorubicin	Glioma	Doxorubicin	Basic research	[40]
2	PTX-encapsulated hyaluronic acid-poly (D,L-lactide-co-glycolide) polymeric micelles (PTX/HA-PLGA micelles)	Glioma	PTX	Basic research	[41]
3	Human umbilical cord blood MSCs loaded with the novel oncolytic adenovirus carrying interleukin (IL)-24 and/or endostatin	Glioma	IL-24	Basic research	[45]
4	Oncolytic virus, CRAd.S.pK7, encapsulated within MSCs	Glioma	CRAd.S.pK7	Basic research	[48]
5	MSCs encoding three critical genes for dopamine synthesis	Parkinson’s disease	Dopamine	Basic research	[54]
6	MSC priming with α-synuclein	Parkinson’s disease	miR 376-3p	Basic research	[55]
7	MSCs naturally possessing matrix metalloproteinase-2 (MMP-2)	Parkinson’s disease	MMP-2	Basic research	[56]
8	Transplantation of MSCs	Alzheimer’s disease	IL-10	Basic research	[65]
9	Bone marrow-derived MSCs	Alzheimer’s disease	Th2 cytokines	Basic research	[66]
10	Bone marrow-derived MSCs	Alzheimer’s disease	miR-146a	Basic research	[67]
11	Mitochondrial Rho-GTPase 1 (Miro1)-overexpressed multipotent MSCs	Stroke	Mitochondria	Basic research	[73]
12	Programmed cell death-ligand 1 (PD-L1) and AKT-modified umbilical cord-derived MSCs	Stroke	PD-L1 and AKT	Basic research	[74]
13	Three-dimensional (3D) spheroid cultured MSCs	Stroke	Unknown	Basic research	[75]
14	Genetically engineered MSCs overexpressing IL-10	Traumatic brain injury	IL-10	Basic research	[77]
15	Engineered MSCs overexpressing BDNF	Traumatic brain injury	BDNF	Basic research	[78]
16	MSCs overexpressing fibroblast growth factor 21 (FGF21)	Traumatic brain injury	FGF21	Basic research	[79]
17	Ro-31-8425-loaded MSCs	Multiple sclerosis	Ro-31-8425	Basic research	[85]
18	MSCs as a carrier of nanoparticles containing biologically active substances	CNS disease	Arbitrary substances	Under analysis in Tashima lab	-

## Data Availability

Data available in a publicly accessible repository. The data presented in this study are openly available in References below. ClinicalTrials. govIdentifier can be found at https://clinicaltrials.gov/ (accessed on 1 January 2024).
